# Expression of claudin, paxillin and FRA-1 in non-nodular breast lesions in association with microcalcifications

**DOI:** 10.1590/S1516-31802013000100016

**Published:** 2013-04-01

**Authors:** José David Kandelman, Angela Flávia Logullo Waitzberg, Jacob Szejnfeld, Ricardo Luiz Smith

**Affiliations:** I MD, PhD. Coordinator of the Mastology Department in CURA Laboratory and Researcher in the Department of Morphology, Universidade Federal de São Paulo (Unifesp), São Paulo, Brazil.; II MD, PhD. Adjunct Professor, Department of Pathology, Universidade Federal de São Paulo (Unifesp), São Paulo, Brazil.; III MD, PhD. Associated Professor, Department of Radiology, Universidade Federal de São Paulo (Unifesp), São Paulo, Brazil.; IV MD, PhD. Chief Professor, Department of Morphology, and Vice-Rector, Universidade Federal de São Paulo (Unifesp), São Paulo, Brazil.

**Keywords:** Mammography, Breast, Cross-sectional studies, Claudins, Paxillin, Mamografia, Mama, Estudos transversais, Claudinas, Paxilina

## Abstract

**CONTEXT AND OBJECTIVE:**

The possible role of adhesion molecules in early breast carcinogenesis has been shown in the literature. We aimed to analyze early adhesion imbalances in non-nodular breast lesions and their association with precursor lesions, in order to ascertain whether these alterations exist and contribute towards early carcinogenesis.

**DESIGN AND SETTING:**

Retrospective cross-sectional study based on medical records at a private radiological clinic in São Paulo, Brazil.

**METHODS:**

We retrospectively reviewed the medical records of all consecutive women attended between August 2006 and July 2007 who presented mammographic evidence of breast microcalcifications classified as Breast Imaging Reporting and Data System Atlas (BI-RADS) type 4. These women underwent stereotaxic biopsy. Clinical, radiological and pathological data were collected, and immunohistochemical assays searched for claudin, paxillin, FRA-1 and HER-2.

**RESULTS:**

Over this period, 127 patients were evaluated. Previous BI-RADS diagnoses showed that 69 cases were in category 4A, 47 in 4B and 11 in 4C. Morphological assessment showed benign entities in 86.5%. Most of the benign lesions showed preserved claudin expression, associated with paxillin (P < 0.001). Paxillin and HER-2 expressions were correlated. FRA-1 expression was also strongly associated with HER-2 expression (P < 0.001).

**CONCLUSIONS:**

Although already present in smaller amounts, imbalance of adhesion molecules is not necessarily prevalent in non-nodular breast lesions. Since FRA-1 expression reached statistically significant correlations with radiological and morphological diagnoses and HER-2 status, it may have a predictive role in this setting.

## RESUMO

**CONTEXTO E OBJETIVO:**

A literatura tem mostrado a importância de moléculas de adesão na carcinogênese precoce de mama. Objetivamos analisar desequilíbrios precoces de adesão em lesões não nodulares da mama e associação com lesões precursoras, a fim de verificar se essas alterações existem e contribuem com a carcinogênese.

**TIPO DE ESTUDO E LOCAL:**

Estudo retrospectivo baseado em prontuários médicos, numa clínica radiológica privada em São Paulo, Brasil.

**MÉTODOS:**

Revisamos retrospectivamente prontuários de todas as mulheres consecutivamente atendidas com evidência mamográfica de microcalcificações mamárias, classificadas como tipo 4 do Breast Imaging Reporting and Data System Atlas (BI-RADS) entre agosto de 2006 e julho de 2007. Elas foram submetidas a biópsia estereotáxica. Dados clínicos, radiológicos e histopatológicos foram coletados e ensaios de imunoistoquímica procuraram por claudina, paxilina, HER-2 e FRA-1.

**RESULTADOS:**

No período, 127 pacientes foram avaliadas. Diagnósticos de BI-RADS anteriores tinham 69 casos na categoria 4A, 47 em 4B, e 11 em 4C. A avaliação morfológica mostrou entidades benignas em 86,5%. A maioria das lesões benignas mostrou expressão preservada de claudina, associada a paxilina (P < 0,001). Expressões de paxilina e HER-2 foram correlacionadas. Expressão de FRA-1 associou-se à de HER-2 (P < 0,001).

**CONCLUSÕES:**

Embora já presente em menor quantidade, o desequilíbrio de moléculas de adesão não é necessariamente prevalente em lesões mamárias nodulares e talvez a expressão de FRA-1 possa ter um papel preditivo neste cenário, uma vez que atingiu correlações estatisticamente significativas com o diagnóstico radiológico e morfológico e com o status de HER-2.

## INTRODUCTION

Precursor lesions of the breast are considered to be entities with high potential to progress toward neoplastic transformation, but they lack the ability to invade and metastasize and, in this sense, are premalignant.^1^ This broad concept includes most of the diagnostic categories exhibiting atypia, such as atypical ductal hyperplasia (ADH), atypical lobular hyperplasia (ALH), ductal carcinoma in situ (DCIS) and lobular carcinoma in situ (LCIS). Although presence of atypia alone is a robust marker for association with cancer,^2^^,^^3^^,^^4^ some other lesions not necessarily harboring atypia or hyperplasia, such as microglandular adenosis and papillary lesions, have also been associated with invasive carcinoma.^5^^,^^6^^,^^7^ Recently, microarray analysis on the triple negative subtype of ductal invasive carcinomas has indicated that actually there is a subset of these cases that does not express claudins. These cases were described as “claudin-low” and were characterized by statistically significantly worse prognosis.^8^

Approximately 23 proteins have already been described as members of the claudin family, and these are essential for the tight junctions (TJs) that form between epithelial cells and between endothelial cells.^9^ They play crucial roles in controlling paracellular transport and in maintaining cell polarity.^10^ These findings shed light on the importance of adhesion molecules and their possible role in early breast carcinogenesis. Loss of claudin 4 has already been reported in cases of lobular in situ carcinoma.^11^

However, cell adhesion is not limited to the claudin system. Several complex molecular schemes contribute to cell adhesion, such as cadherins, integrins and CD4, which have previously been studied in relation to breast cancer. Other than these important components of the cell adherence system, some novel mechanisms are directly or indirectly involved in cell adhesion, cell stability and prevention of cell migration. Focal adhesion sites contain multiple structural proteins such as talin, paxillin and focal adhesion kinase (FAK).^12^ Paxillin is an adaptor protein with an important role in cell spreading and motility,^13^ and is located in the region of cell contact with the underlying extracellular matrix. In the physiological state, it functions as an adaptor protein that recruits several cytoskeleton and signaling proteins into a complex, thereby enabling transmission of coordinated downstream signals.^14^ Tyrosine phosphorylation of paxillin has been observed following integrin-dependent cell adhesion to extracellular matrix proteins, thus implicating paxillin in integrin-mediated signaling and focal adhesion formation.^14^^,^^15^ Few reports have described paxillin distribution in breast tissue and ductal carcinomas.

In addition, while mediating cell adhesion, many cell adhesion molecules that have already been described act as tumor suppressors. Disrupted cell-cell or cell-extracellular matrix (ECM) adhesion significantly contributes towards uncontrolled cell proliferation and progressive distortion of normal tissue architecture.^16^ In this regard, another molecular pathway involved in cell adhesion, motility and invasion, with a potential role in breast cancer, is the AP-1 FRA-1 family.^17^^,^^18^ A recent study has shown that high frequency of FRA-1 in ductal carcinoma in situ may be associated with early events in breast carcinogenesis, since the frequency of FRA-1 expression in invasive cancer was lower than the frequency of these in situ lesions.^19^ Interestingly, there are several reports in the literature regarding interactions between HER-2 expression (one of the most important receptor kinase growth factors in breast cancer) and adhesion molecules. HER-2 overexpression has previously been reported to be associated with claudin 4^20^ and paxillin expression^21^ in breast carcinomas, which led us to further investigate whether, in our set of non-nodular breast lesions, this association would be confirmed.

## OBJECTIVES

Our aim was therefore to analyze possible early adhesion imbalances by assessing these markers (claudin, paxillin, HER-2 and FRA-1) in non-nodular breast lesions and their possible association with precursor lesions, in order to ascertain whether these alterations might be present and contribute towards early carcinogenesis.

## METHODS

The medical records of all consecutive women attended between August 2006 and July 2007 who presented non-palpable primary breast lesions with mammographic evidence of breast microcalcifications and clinically suspicions were identified from the archives of a private radiological diagnostic clinic (CURA Diagnostics, São Paulo, Brazil), and retrospectively reviewed. The patients were referred for further core biopsy or vacuum-assisted biopsy sampling, and their radiological initial and final classifications were registered. The Ethics Committee of the Federal University of São Paulo (Universidade Federal de São Paulo, Unifesp) approved this study (protocol CEP 0002/08). The patients gave their informed consent for the diagnostic procedures before inclusion in this retrospective study. The inclusion criteria were that the patients should present primary non-nodular breast lesions, previously classified as Breast Imaging Reporting and Data System Atlas (BI-RADS) type 4, and have undergone further radiological diagnosis, with morphological sampling for surgical pathological diagnosis. All the cases were objectively analyzed by two observers (trained radiologists). In the event of discordance regarding the classification, they worked to reach a consensus. Patients with histories of preoperative treatment with chemotherapy or radiotherapy for other reasons were excluded. Only the patients whose paraffin blocks and clinical data were available for further analysis were included. Clinical, radiological and morphological data were collected. Morphological diagnoses were assessed in accordance with World Health Organization (WHO) guidelines and grouped as benign, premalignant or malignant (ductal carcinoma in situ and invasive carcinoma).

### Biopsy method

Stereotaxic vacuum-assisted core biopsy was performed for histopathological analysis, using an 11-gauge needle and a vacuum-assisted breast biopsy device (Mammotome Biopsy System, Johnson & Johnson Ethicon Endo-Surgery), under local injection anesthesia (lidocaine in association with 2% epinephrine). The patient was positioned according to the location of the targeted breast calcifications, always attempting to use the shortest path to reach the target area. X-rays were produced before and after obtaining specimens. Needle insertion was aimed towards the microcalcifications and, in cases of multiple foci, the most suspicious group according to the radiological appearance was chosen. The radiologist sought to obtain 15 to 18 specimens for histopathology. After concluding the biopsy procedure, a titanium clip was inserted, thus marking the biopsied area. Before being discarded, the needle was washed and the material remaining in the tubes was collected and subjected to further centrifugation.

### Morphological assessment

All the specimens were identified and properly labeled with the patient’s data. The samples were radiographed to ascertain whether microcalcifications were present, and were packaged in vials with 10% buffered formalin. The specimens were then processed and embedded in paraffin blocks, and slides of thickness 5 microns were obtained and stained with hematoxylin-eosin. Further sections were subjected to immunohistochemical assays.

On morphological examination, the following categories of benign lesions were noted: changes associated with benign fibrocystic breast disease, fibroadenomas, papillomas and benign tumors such as lipoma and adenomyolipoma. Proliferative lesions were reported as typical or atypical hyperplasia. Malignant lesions were classified as in situ, microinvasive or infiltrative carcinomas. Along with the histological diagnosis, the histological grade and percentage of the sample affected by the lesion were reported. A single experienced pathologist examined all the cases and was blinded to the imaging results (AFL).

### Immunohistochemistry

Control tissues were included in each reaction. Whole 5-mm tissue sections from each block were subjected to each essay (for claudin, paxillin, HER-2 and FRA-1), cut and transferred to silanized slides, and left to dry overnight at 56 ºC. The next day, the slides were dewaxed in xylene, rehydrated in graded alcohol and washed with water. Antigen retrieval was performed using a pressure cooker and 10 mM citrate buffer (pH 6.0). The samples were quenched with 6% hydrogen peroxide and incubated overnight at 4 ºC with different antibodies, to be examined using immunohistochemistry. The following day, the slides were rinsed with PBS and incubated with the secondary antibody, for 30 min at 37 ºC. The slides were rinsed again with phosphate buffered saline (PBS) and incubated with polymer (Novolink Max Polymer cat# RE7260-K, Novocastra Lab, Newcastle upon Tyne, UK) for 30 min at 37 ºC. The slides were developed with 100 mg% DAB as the chromogen, with 0.06% hydrogen peroxide, and counterstained with Harris hematoxylin. Positive and negative control slides were included. The negative control was a slide from which the primary antibody had been omitted. Table 1 presents the primary antibodies and dilution rates utilized. All reactions were performed in the Pathology Department of A. C. Camargo Hospital, São Paulo.

**Table 1.** Antibodies, clones and titers used in the immunohistochemical assay

Antibodies	Clones	Titers	Producer
FRA-1 (C12)	Monoclonal in mice	1:50	Santa Cruz, cat# sc28310, Santa Cruz, CA, USA
Claudin-4	Polyclonal in rabbit	1:100	Affinity Bioreagents, cat# PA1-20906, Golden, CO, USA
Paxillin	Monoclonal in mice 5H11	1:400	LabVision, cat# MS404, Fremont, CA, USA

Claudin-4, paxillin and HER-2 membrane expression were assessed by identifying ductal cells from their chicken-wire pattern staining, and were classified using the standard HER-2 classification system of Herceptest, as 1 to 3+. FRA-1 showed nuclear-exclusive and diffuse staining. Lesions were defined as positive if at least 10% of the true neoplastic tumor cells expressed the protein.

### Radiological assessment

The initial mammograms brought by patients from other services, including the BI-RADS 4 classification, were reviewed. All the cases were analyzed by two observers (trained radiologists) and were objectively reclassified in accordance with the fourth edition of BI-RADS. In the event of discordant classification, the two observers worked together to reach a consensus.

### Statistical analysis

Spearman’s rank test was used to estimate the relationships between staining patterns of different antibodies. The correlation between antigen expression and other parameters was studied using Pearson’s chi-square or Fisher’s exact test. The numbers of false-positive and false-negative mammograms and the number of examinations with full agreement between the BI-RADS (fourth edition) classification and the histopathological findings were calculated. Associations between clinical, pathological and radiological variables were evaluated using Fisher’s exact test or the chi-square test, as appropriate. Two-sided P values less than 0.05 were considered statistically significant. All the analyses were carried out using the SAS 9.1 software (Statistical Analysis System, Cary, NC, USA).

## RESULTS

During the study period, 127 patients were evaluated and fulfilled the inclusion criteria for this study. Among these 127 patients, the previous BI-RADS diagnoses included 69 cases of category 4A, 47 of 4B, and 11 of 4C. The histopathological evaluation defined the majority of the cases included (86.5%) as benign entities. These included 37 cases of nonspecific functional and cystic alterations, one case of papilloma, one case of fibroadenoma and 28 cases of usual ductal hyperplasia. Atypical hyperplasia was found in five cases and carcinoma (in situ or invasive) was present in the remaining 16 cases. The distribution of these results is detailed in Table 2 (above).

**Table 2.** Histopathological and radiological diagnoses of 127 samples from non-nodular breast lesions

Variable	BI-RADS		Total	P-value
	4A	4B or 4C		
Histopathological diagnosis				
Benign	62 (89.9%)	44 (75.9%)	106 (83.5%)	0.0387
ADH (atypical ductal hyperplasia)	3 (4.3%)	2 (3.4%)	5 (3.9%)	
Malignant (in situ and invasive)	4 (5.8%)	12 (20.7%)	16 (12.6%)	
Total	69 (100%)	58 (100%)	127 (100%)	

BI-RADS = Breast Imaging Reporting and Data System Atlas.

Most of the benign lesions showed preserved claudin-4 expression (Table 3). In normal breast tissue or mammary glands presenting mild dysfunctional alterations such as fibroadenosis or fibrosclerosis, claudin expression was present in ductal cells, with a membrane pattern comparable to HER-2 staining. Usually, all mature ductal cells are positive, but the staining is not uniformly distributed, since some cells are strongly positive and others only moderately so.

**Table 3.** Frequencies of FRA-1, claudin and paxillin in 127 non-nodular breast lesions

Variable	Total
FRA-1	
Negative	76 (598%)
Positive	51 (40.2%)
Total	127 (100.0%)
HER-2	
Negative	109 (85.8%)
Positive	18 (14.2%)
Total	127 (100.0%)
Claudin	
Negative	18 (14.2%)
Positive	109 (85.8%)
Total	127 (100.0%)
Paxillin	
Negative	62 (48.,8%)
Positive	65 (51.2%)
Total	127 (100.0%)

We found the normal tissue pattern commonly equivalent to category 2+ of the ASCO/USCAP (American Society of Clinical Oncology/United States and Canadian Academy of Pathology) classification for HER-2 expression, with up to 30% of the cells strongly positive, among the other 70% with discontinuous positivity. By observing the scope of different alterations on the benign diagnostic samples, we could see that apocrine metaplasia was almost always totally negative for claudin-4 and extended areas of adenosis were usually negative. On the contrary, microglandular adenosis was strongly positive. Atrophic cells from cystic ducts or dysfunctional postmenopausal women are also negative.

We also noticed that lactation-type alteration was negative for claudin-4 and, amazingly, within the papilloma present in the study, claudin-4 expression was confined to the surface epithelia, while the bulk and inner layer were negative. Columnar alterations are usually negative for claudin-4, but the few examples of flat atypia in our samples showed a markedly 3+ pattern of claudin-4 expression.

**Figure 1.**
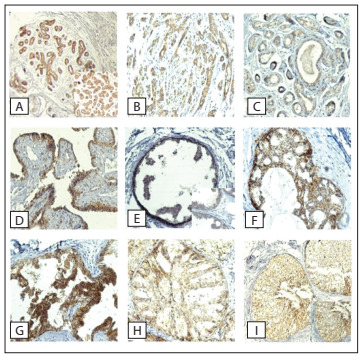
Claudin-4 immunoexpression in non-nodular breast lesions: (A) ductal lobular terminal unit; (B) fibrosclerosis; (C) attenuation in fibroadenosis; (D) papilloma; (E) hyperplasia of usual type; (F and G) atypical hyperplasia; and (H and I) ductal carcinoma in situ.

The usual type of hyperplasia showed variable results regarding claudin-4 expression. Most of the cases were positive, but the intensity and percentage of positive cells were usually lower than in cases with atypia present. As in the few cases harboring foci of flat atypia, atypical ductal hyperplasia showed strong claudin-4 expression, along with the majority of the ductal carcinoma in situ samples. All but one of the atypical and malignant cases were positive for claudin-4 (20/21), although without reaching statistical significance for morphological or radiological diagnosis (Table 4). Figure 1 shows a strongly 3+ positive claudin-4 ductal carcinoma in situ case.

**Table 4.** Correlation of HER-2, paxillin, claudin and FRA-1 results with morphological and radiological variables

Variable	BI-RADS		Total	P-value	Morphological assessment		Total	P-value
	4A	4B or 4C			Benign	Atypical malignant		
FRA-1								
Negative	48 (69.6%)	28 (48.3%)	76 (59.8%)	0.0241	68 (64.2%)	8 (38.1%)	76 (59.8%)	0.0475
Positive	21 (30.4%)	30 (51.7%)	51 (40.2%)		38 (35.8%)	13 (61.9%)	51 (40.2%)	
Total	69 (100%)	58 (100%)	127 (100%)		106 (100%)	21 (100%)	127 (100%)	
HER-2								
Negative	64 (92.8%)	45 (77.6%)	109 (85.8%)	0.0288	96 (90.6%)	13 (61.9%)	109 (85.8%)	0.0020
Positive	5 (7.2%)	13 (22.4%)	18 (14.2%)		10 (9.4%)	8 (38.1%)	18 (14.2%)	
Total	69 (100%)	58 (100%)	127 (100%)		106 (100%)	21 (100%)	127 (100.0%)	
Claudin								
Negative	9 (13%)	9 (15.5%)	18 (14.2%)	0.8865	17 (16%)	1 (4.8%)	18 (14.2%)	0.3040
Positive	60 (87%)	49 (84.5%)	109 (85.8%)		89 (84%)	20 (95.2%)	109 (85.8%)	
Total	69 (100%)	58 (100%)	127 (100%)		106 (100%)	21 (100%)	127 (100.0%)	
Paxillin								
Negative	31 (44.9%)	31 (53.4%)	62 (48.8%)	0.4362	53 (50%)	9 (42.9%)	62 (48.8%)	0.7190
Positive	38 (55.1%)	27 (46.6%)	65 (51.2%)		53 (50%)	12 (57.1%)	65 (51.2%)	
Total	69 (100%)	58 (100%)	127 (100%)		106 (100%)	21 (100%)	127 (100.0%)	

Since the association between claudin-4 expression and HER-2 overexpression had previously been reported in breast carcinomas,^20^ we investigated whether this association would be confirmed in our set of non-nodular breast lesions. We found that HER-2 expression marginally correlated with claudin-4 (P = 0.0734; Table 5).

**Table 5.** Correlation of HER-2, paxillin, claudin and FRA-1 results

Variables	HER-2		Total	P-value	Paxillin		Total	P-value
	Negative	Positive			Negative	Positive		
FRA-1								
Negative	72 (66.1%)	4 (22.2%)	76 (59.8%)	0.0011	42 (55.3%)	34 (44.7%)	76 (100%)	0.013
Positive	37 (33.9%)	14 (77.8%)	51 (40.2%)		20 (39.2%)	31 (60.8%)	51 (100%)	
Total	109 (100%)	18 (100%)	127 (100%)		62 (48.8%)	65 (51.2%)	127 (100%)	
Claudin								
Negative	18 (16.5%)	0 (0%)	18 (14.2%)	0.0734	18 (29%)	0 (0%)	18 (14.2%)	< 0.001
Positive	91 (83.5%)	18 (100%)	109 (85.8%)		44 (71%)	65 (100%)	109 (85.8%)	
Total	109 (100%)	18 (100%)	127 (100%)		62 (100%)	65 (100%)	127 (100%)	
Paxillin								
Negative	56 (51.4%)	6 (33.3%)	62 (48.8%)	0.2443				
Positive	53 (48.6%)	12 (66.7%)	65 (51.2%)					
Total	109 (100%)	18 (100%)	127 (100%)					

**Figure 2.**
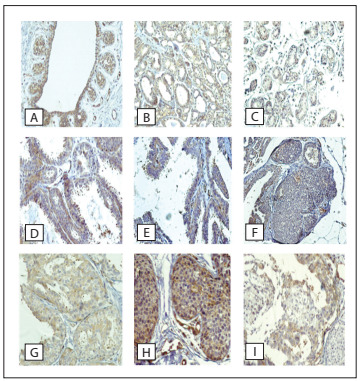
Paxillin immunoexpression in non-nodular breast lesions: (A) ductal lobular terminal unit; (B) fibrosclerosis; (C) attenuation in fibroadenosis; (D) columnar alteration; (E) papilloma; (F) hyperplasia of usual type; (G) atypical hyperplasia; and (H and I) ductal carcinoma in situ.

Among the other proteins evaluated, claudin 4 expression was associated with paxillin (P < 0.001; Table 4). Paxillin expression was detected uniformly in all normal breast tissue (Figure 2). Its distribution correlated with the epithelial layers, such that the basal cells tended to be more positive than the luminal elements. However, the pattern was similar to that of HER-2 expression, i.e. more staining that was more membrane-based than cytoplasmic. In preserved breast elements, paxillin expression was delicate, and finer than the chicken-wire membrane-based HER-2 pattern. The basal membrane was always positive, as were the myoepithelial cells. Some functional alterations like apocrine metaplasia and columnar alterations showed diminished or absent paxillin expression, and adenosis and fibrosclerosis tended to maintain paxillin but in a rather diffuse and weak cytoplasmic pattern, with fewer membrane reactive spots. Presence of atypia conferred stronger and homogeneous evidence of paxillin in affected cells. Paxillin expression did not correlate with the morphological or radiological results (Table 4). It did not correlate with HER-2 expression (P = 0.013) or with FRA-1 (P = 0.2443, Table 5).

**Figure 3.**
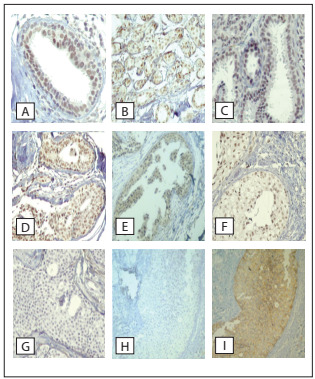
FRA-1 immunoexpression in non-nodular breast lesions: (A) ductal lobular terminal unit; (B) fibrosclerosis; (C) columnar alteration; (D) hyperplasia of usual type; (E) atypical ductal hyperplasia; (F and G) positive and negative ductal carcinoma in situ; and (H and I) same area of ductal carcinoma in situ, negative for FRA-1 and positive for HER-2 expression.

FRA-1 expression was always evident around ductal structures, thus strongly marking the basal membrane (Figure 3). Ductal cells were usually negative, but in normal ductal structures, some scattered elements showed nuclear-exclusive staining. In one third of the cases with normal breast tissue, more than 10% of the preserved ductal cells expressed FRA-1. However, the majority of non-proliferative lesions, including papillary lesions, adenosis, apocrine metaplasia and columnar alterations remained diffusely negative. The reactivity to FRA-1 tended to be more intense and diffusely distributed in atypical and malignant lesions (13 out of 21 cases), with positive associations with atypia and malignancy (P = 0.04, Table 4). FRA-1 expression was also strongly associated with HER-2 expression (P < 0.001; Table 5).

## DISCUSSION

Analysis on non-nodular lesions of the breast encompasses a large range of entities since, aside from the premalignant entities, many benign lesions and alterations may present microcalcifications over time. Vacuum-assisted or core biopsies are usually indicated in cases that are radiologically classified as BI-RADS 4. According to the BI-RADS classification, this category includes a wide range of lesions: category 4A is defined as “findings needing intervention with a low suspicion of malignancy”; category 4B includes “lesions with an intermediate suspicion of malignancy” and the new category 4C consists of lesions with “findings of moderate concern, but not classic for malignancy”.^22^ Interestingly, the results from the morphological diagnosis were statistically associated with these BI-RADS categories at the same non-nodular lesions, and this became more significant when categories B and C were merged together. Since the adhesion properties of epithelial mammary cells are linked to basal membrane status and also to the surrounding extracellular matrix, non-nodular lesions seem to be an ideal vehicle for pinpointing early events in stromal and epithelial cells that may only have acquired subtly compromised adhesion properties.

We also had the opportunity to investigate the expression of these proteins in many specimens of virtually nearly normal breast tissue, which were collected and fixed in accordance with the best recommendations. These provided a good description of claudin, FRA-1 and paxillin distribution in non-proliferative lesions, as well as in nearly normal breast tissue.

In this regard, we found some imbalance of adhesion protein expression even in the benign cases. Claudin-4 expression, for instance, was negative in 17 cases of non-malignant breast lesions, especially those harboring apocrine metaplasia and adenosis. Another important finding was that claudin-4 expression was maintained through the progression to atypical lesions and in situ carcinomas, thus resulting in a larger contingent of positive samples in our series and therefore lacking correlation with morphological or radiological categories. Since the recent findings of loss of claudin expression have mostly been linked to estrogen-negative and high-grade carcinomas^20^ and/or aggressive triple negative cases, it can be inferred that claudin-4 expression imbalance is related most exclusively to precursor lesions of highly aggressive tumors such as high-grade ductal carcinoma in situ.^23^ It seems that these events were not sampled in our series, or that they occur later on in breast carcinogenesis.

FRA-1 expression in normal and non-neoplastic breast tissue showed nuclear immunoreactivity, as reported by Song et al.^24^ We did not find any example of cytoplasmic reactivity for FRA-1, even at in our single case of invasive carcinoma. This may be explained by the fact that this particular case was a mucinous well-differentiated carcinoma. However, we found a shift between benign and malignant lesions, particularly when atypical cases were merged with in situ carcinomas. While one third of the benign cases were FRA-1 positive, over 61% of the malignant cases showed some nuclear reaction to FRA-1 (P = 0.0475), concordant with the findings of Song et al.^24^

Interestingly, FRA-1 expression also correlated with BI-RADS classification. The possible role of FRA-1 in early carcinogenesis was previously demonstrated when, in a larger series of in situ and invasive nodular cases, the frequency of FRA-1 expression in invasive cancer was lower than it was in in situ lesions.^19^ It seems that in situ carcinomas harbor the highest possible amount of FRA-1 nuclear expression and, according to other reports,^24^^,^^25^ the invasion process may be accompanied by concomitant detection of cytoplasmic FRA-1 and diminution of FRA-1 expression, in comparison with in situ cases. One possible explanation for FRA-1 detection in cytoplasm could be that extracellular secretion of FRA-1 may occur, internalized by certain neoplastic cells, given that the tumor-associated macrophages that are present in invasive tumors intensively express FRA-1 and therefore indirectly support invasion and progression of carcinomas cells.^26^ Alternatively, the higher frequency of FRA-1 expression in membrane-restricted lesions should be addressed, in the light of reports on impressive FRA-1 effects relating to invasiveness, cell motility, aggressiveness and regulation of proteins implicated in tumor progression, which have been described in cultured breast tissue cells.^27^ It is possible that the momentum shortly before the impressive shift from in situ to invasive carcinoma, with focal infiltration, constitutes the most demanding situation for nuclear FRA-1 production.

In normal mammary cells, paxillin had previously seldom been described in humans, and the few reports available are rather similar to what we have reported here.^12^ On the other hand, invasive ductal carcinomas showed expression of 27.7% (short S) to 50%.^12^ We were able to describe paxillin expression with a wider range of functional alterations, and half of the benign cases were positive. In contrast to another report,^12^ we did not find any association between paxillin expression and progression to malignancy or BI-RADS classification. However, when assessed in cytological smears, paxillin failed to reach any concordance with invasion or prognostic variables.^28^ It remains unclear whether paxillin may be associated with invasion, since it regulates focal adhesion kinase (FAK) function,^29^ which is a marker of malignant transformation rather than invasion.^30^ A substantial set of malignant and premalignant cases needs to be evaluated in order to provide further responses to this question, since paxillin expression was correlated with claudin and FRA-1 expression in our cases (P < 0.001 and 0.013, respectively).

It appears that adhesion signals and complex cellular protein complexes are somehow interrelated, and that subtle imbalance of one settlement may interfere with others. Alternatively, it is possible that cellular signaling aiming towards switching the adhesion status is launched in a coordinated manner, which may affect some, if not all, adhesion complexes in a cascade.

There are several reports in the literature regarding interactions between HER-2 expression and adhesion molecules. Paxillin expression was found to correlate with HER-2 gene amplification in 314 cases of invasive carcinoma, which led to speculation about whether paxillin might be a marker that could influence the predictive value of HER-2 regarding the response to adjuvant treatment.^21^ We could not identify such a correlation, since the majority of our cases comprised benign and negative HER-2 samples, concordant with the findings of Madan et al.^12^ Claudin-4 was found to correlate with HER-2 immunohistochemical expression in 299 cases of invasive ductal carcinoma,^20^ but another report did not find this correlation in 412 tumors.^31^ In our series, this correlation was also not found (P = 0.0734). It seems that the larger contingent of non-neoplastic cases impeded a more specific correlation between these two variables.

Finally, we were able to report a statistically significant correlation between HER-2 and FRA-1 expression (P < 0.001). Since both of these variables also statistically correlated with the radiological and morphological results in this set of non-nodular breast lesions, a larger series of premalignant and malignant cases is essential in order to clarify the possible significance of these findings.

## CONCLUSION

We conclude that, although already present in smaller amounts, imbalance of adhesion molecules is not necessarily prevalent in non-nodular breast lesions. Moreover, since FRA-1 expression reached statistically significant correlations with radiological and morphological diagnoses and with HER-2 status, perhaps this expression should be evaluated in a larger series, in order to investigate its potential predictive role in this setting.
